# Identification and characterization of major histocompatibility complex class IIB alleles from three species of European ranid frogs

**Published:** 2014-12

**Authors:** Béla A. Marosi, Karen M. Kiemnec-Tyburczy, Ioan V. Ghira, Tibor Sos, Octavian Popescu

**Affiliations:** 1 Department of Parasitology, University of Agricultural Science and Veterinary Medicine, Mănăștur Street nr. 3-5, RO-400372 Cluj-Napoca, Romania, marosib@yahoo.com; 2 Department of Biological Sciences, Humboldt State University 95521 USA, kmk877@humboldt.edu; 3 Faculty of Biology and Geology, Babes-Bolyai-University Cluj-Napoca, Clinicilor Street 5-7, RO-400006 Cluj-Napoca, Romania, ighira2002@yahoo.com; 4 Association for Bird and Nature Protection “Milvus Group”, Targu Mures, 22 Crinului Street, RO-540343, Romania, tibor.sos@gmail.com; 5 Molecular Biology Center, Interdisciplinary Research Institute on Bio-Nano-Sciences, Babes-Bolyai University Cluj-Napoca, 42 Treboniu Laurian Street, RO-400271 Cluj-Napoca, Romania, opopescu.ubbcluj@gmail.com

**Keywords:** Antigen Binding Site, Anura, *Pelophylax*, *Rana*, *Ranidae*

## Abstract

Immune genes of the major histocompatibility complex (MHC) are among the most polymorphic genes in the vertebrate genome. Due to their polymorphic nature, they are often used to assess the adaptive genetic variability of natural populations. This study describes the first molecular characterization of 13 partial MHC class IIB sequences from three European ranid frogs. The utility of previously published primers was expanded by using them to successfully amplify eight exon 2 alleles from *Rana arvalis*.We also designed a novel primer set that successfully amplified exon 2 from *Pelophylax kurtmuelleri*. *Pelophylax lessonae* was also designed as part of this study. Results indicate the presence of one or two class IIB loci in these three species. In *R. arvalis*, significant evidence of positive selection acting on MHC antigen binding sites was found. Many European ranid populations are experiencing disease-related declines; the newly developed primers can, therefore, be used for further population analyses of native frogs.

## INTRODUCTION

Major histocompatibility complex (MHC) is a relatively large region found in the genomes of all jawed vertebrates. Most of the genes in this region are necessary for a functional immune response and are thus vital to the survival of an organism. MHC class II proteins, specifically, are expressed on the surface of specialized antigen presenting cells, their primary function being presenting extracellular antigens to helper T cells [1]. The mature MHC class II complex is composed of one alpha (A) and one beta (B) peptide chain encoded by the MHC II A and B genes, respectively. The antigen binding site (ABS) of this heteromeric protein complex is encoded by the second exons of the A and B genes [2]. The amino acid residues in the ABS bind pathogen-derived peptides are often extremely polymorphic and show signatures of natural selection [3]. 

Since MHC genes play such a key role in the vertebrate immune response, they are often used to assess the structure and status of wildlife populations. Interest in monitoring amphibian populations, in particular, is increasing because of the emergence of infectious diseases caused by *Batrachochytrium dendrobatidis* (*Bd*) [4] and ranavirus(es) [5]. Many European ranid frogs are experiencing population declines because of these diseases (e.g., *Rana klepton esculenta* [6] and *Rana temporaria* [7]), but only few studies have characterized MHC genes in European frogs [8, 9]. Evaluation of the genetic variation at MHC loci in endangered and threatened amphibian species may be useful for developing conservation management strategies that target populations highly threatened by infections and/or by the effects of inbreeding [10]. The aim of the present study was to develop ranid-specific primers and to characterize the MHC class IIB exon 2 in three European ranid species: the Moor frog (*Rana arvalis)*, the Balkan Water Frog (*Pelophylax kurtmuelleri)* and the Pool Frog (*Pelophylax lessonae*). We chose to focus our study on MHC class IIB because it has been successfully characterized and shown to be under selection in other ranids. Therefore, characterizing this type of MHC gene facilitates comparisons between our focal species and other ranids.

## MATERIALS AND METHODS

**Specimen collection and** **DNA** **extraction:** We obtained ethanol-preserved tissues from *R. arvalis *(n=3), *P. kurtmuelleri *(n=2),*R. temporaria* (n=2) that were collected in Romania and archived at the Babes-BolyaiUniversityZoologicalMuseum. We collected two *P. lessonae* adults in south Romania near the Danube River. All individuals from all species were collected from different populations. Genomic DNA was extracted from toe clips of all frogs using the Nucleospin®Tissue Kit following the standard protocol (Macherey Nagel, Düren, Germany). Since *P. kurtmuelleri* is morphologically difficult to distinguish from *R. ridibunda*, we isolated approximately 590 bps of the 16S RNA mitochondrial gene using 16Sar and 16Sbr primers [11] and compared it to other sequences available in GenBank to confirm that our individuals were *P. kurtmuelleri*.

***PCR amplification: ***We used a degenerate primer pair (MHC-F and MHC-5R) developed by Hauswaldt et al. [6] to amplify a 235 bp fragment from *R. arvalis* and *R. temporaria* genomic DNA. PCR was carried out with GoTaq® Flexi DNA Polymerase (Promega, Madison, WI) and PCR conditions were made available by the corresponding author upon request. Using the published sequences from other *Rana* [9; 12], we developed a new set of degenerate primers (RanaF 5′-CAGTGTTATTACCGGAACGGGACG-3′ and RanaR2: 5′-TTTSMGSTCTATGGCTGYAGG-3′) that we used to amplify exon 2 from *P. kurtmuelleri* and *P. lessonae*. The samples were run on 2.5% agarose gel and PCR products were extracted from the gel using the Nucleospin® Extract Kit (Macherey Nagel). Purified PCR products were ligated into the pTZ57R/T vector using the InsTAclone™ PCR Cloning Kit (Fermentas, Cluj-Napoca, Romania). XL1 blue *Escherichiacoli* were used for transformation. Plasmid DNA was extracted from 10 positive colonies by GeneJET™ Plasmid Miniprep Kit (Fermetas). After restriction enzyme verification of the size of each vector insert, five positive clones from each individual were sent to Macrogen Inc., (Seoul, Korea) for sequencing.

**Data analysis** **:** We used BioEdit v7.0.9 [13] to edit and trim our sequences. For alignment of the sequences (nucleotide and amino acid), we used MEGA version 5.0 [14]. To estimate the evolutionary divergence between sequences, average pairwise nucleotide distances (Kimura 2-parameter model) and Poisson correction were used. 

To test for positive selection on the putative ABS of *R. arvalis* and *P. lessonae* separately, we used a one-tailed Z-test of selection calculated in MEGA. We determined the ABS residues for our frog sequences according to the model of Tong et al. [15]. The average codon-based evolutionary divergence was analyzed separately for the ABS and non-ABS; the rate of synonymous substitutions (*d*_S_) and rate of nonsynonymous (*d*_N_) substitutions and the differences between synonymous and nonsynonymous distances (*d*_N _- *d*_S_) were estimated using the Nei-Gojobori method with Jukes-cantor correction in MEGA 5.0. Variances were computed using the bootstrap method (1000 replicates). To test for positive selection acting on the entire alignment of all four species (including both ABS and non-ABS), we performed PARRIS [16].

**Figure 1:**
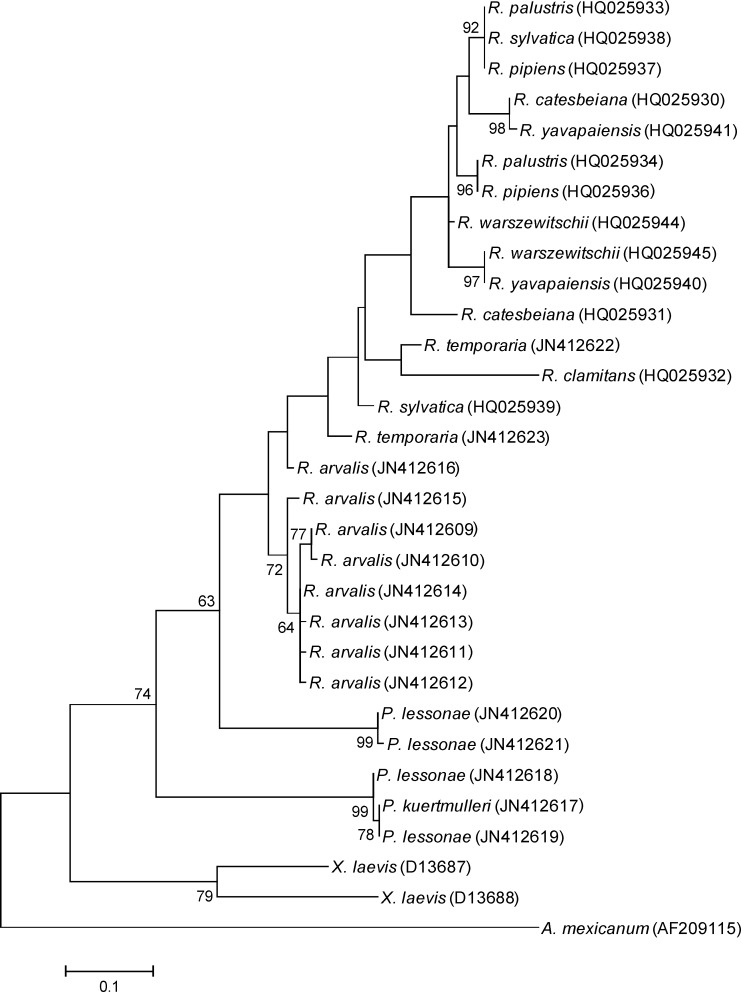
Phylogenetic relationships of the MHC class IIB exon 2 sequences in frogs generated with maximum likelihood (see text for details). *Ambystoma mexicanum* was used as an outgroup and bootstrap values (1000 replicates) are shown for nodes that received greater than 60% support. GenBank accession numbers are given after the sequence name

A phylogenetic tree of anuran MHC IIB DNA sequences was constructed from an alignment of 152 bp, present in multiple anuran sequences (Fig. 1). To determine which model of molecular evolution best fit the data, we used FindModel (available at http://www.hiv.lanl.gov/content/sequence/findmodel/findmodel.html). Evolutionary relationships were inferred using maximum likelihood based on Kimura 2-parameter plus Gamma model with 1000 bootstrap replicates in MEGA.

## RESULTS AND DISCUSSION

We isolated a total of 13 MHC class II beta exon 2 alleles from the genomic DNA of the three focal frog species we sampled (see Fig. 1 for GenBank accession nos.). The translated amino acid sequences of all alleles showed significant amino acid similarity to other frog sequences accessioned in GenBank (BLAST e-values less than 2.4*e^-48^). Using the Hauswaldt et al. [8] primer pair, we amplified eight alleles (186 bp) from three *R. arvalis* individuals. We also isolated two additional alleles from two *R. temporaria* that were longer and not identical to the ones characterized by Zeisset and Beebee [9]. 

 Using our newly developed primer pair (RanaF and RanaR2), we obtained four 196 bp alleles from two *P. lessonae* and one allele from two *P. kurtmuelleri*. We found that the divergence in the deduced amino acid sequences of our sequences varied among species.Amino acid divergence ranged from 0.017 (± 0.012) - 0.053 (± 0.031) and from 0.00-0.016 (± 0.015) within *R. arvalis* and *P. lessonae* individuals, respectively. In *R. arvalis*, out of the 61 amino acids in the alignment, there were 12 variable amino acid positions among the eight alleles. Eight of these variable sites were predicted to be in the ABS based on the model of Tong et al. [15]. In addition, *d*_N_was significantly higher than *d*_S _(Table 1). In contrast, *d*_N_ was not higher than *d*_S_ in sites outside the ABS. In *P. lessonae*, we found 30 variable amino acid sites, seven of them occurring in the ABS and 23 in the non–ABS region. We found no significant evidence of positive selection in the ABS and/or non-ABS in *P. lessonae* (Table 1). The PARRIS analysis of positive selection did not detect any evidence of positive selection acting on the alignment as a whole.

**Table 1:** Estimates of nonsynonymous (*d*_N_) and synonymous (*d*_S_) substitution rates of the MHC class II exon 2 sequences from two ranid frog species

**Sites**	***n***	***d***_S_	***d***_N_	***d***_N_ **-** ***d***_S_	***P***
*R. arvalis *ABS	8	0.015 ± 0.014	0.154 ±0.009	0.139 ± 0.043	0.003
*R. arvalis *Non-ABS	8	0.032 ± 0.016	0.01±0.005	-0.022 ± 0.017	1.0
*P. lessonae *ABS	4	0.324 ± 0.280	0.431 ±0.214	0.107 ± 0.333	0.375
*P. lessonae *Non-ABS	4	0.334 ± 0.121	0.199 ± 0.041	-0.135 ± 0.134	1.0

The phylogenetic analysis indicated common alleles between species (Fig. 1). The* R. arvalis* alleles formed one well-defined clade, but two of the *P. lessonae* alleles grouped together with the *P. kurtmuelleri* sequence with very high support. Notably, the two other *P. lessonae* alleles formed a separate clade that was more closely related to the New World ranid species. Surprisingly, the *R. temporaria* alleles we amplified grouped together with MHC sequences from New World*Rana* sequences, although the node separating *R. temporaria* and New World ranids from the *R. arvalis* sequences had low support. 

In our study, we presented the first molecular characterization of the MHC class IIB exon 2 sequences from three European ranid species: *R. arvalis*, *P. kurtmuelleri* and *P. lessonae*. Although we sampled a fairly small number of individuals, the number of alleles we isolated and identified was relatively high and we found evidence of positive selection acting on the ABS in *R. arvalis*, a pattern that has been found in other anuran MHC characterization studies [9]. Although we did not detect positive selection in *P. lessonae* or on the alignment of all sequences as a group, we suspect that this result has occurred due to small sample size rather than a true lack of historical selection. As more data is gathered from these species, more comprehensive tests of selection can be used to better assess the effects of selection.

Other studies have identified MHC class II alleles under selection in other European ranid frog species [8,9]. In addition, polymorphism at class IIB loci is commonly observed. For example, Zeisset and Beebee [9] found eight alleles in 215 *R. temporaria* individuals and May and Beebee [17] isolated five alleles from 200 *Bufo calamita*. In contrast, eight alleles were found in 20 individuals sampled from multiple populations of *Bombina bombina* [8]. Thus, although our sample sizes are relatively small, the number of alleles we characterized in *P. lessonae* and *R. arvalis* is similar to what has been found in other studies of anuran MHC class IIB diversity.

We infer that the *R. arvalis* genome has least two MHC class IIB loci because we recovered four unique alleles from one individual. We also predict that *P. lessonae* has at least two loci, because the number of amino acid differences between two distinct groups of alleles isolated from this species is much larger than those between sequences isolated from other species. The amino acid distances between the two individuals were high (0.654) while distances between alleles within individuals were low (0.000 and 0.020). The presence of two loci may also explain why the four *P. lessonae* alleles did not group together in the phylogenetic tree. 

European frogs are predominately threatened by two major emerging infectious agents, *Bd* and ranavirus [18]. We found evidence of positive selection in *R. arvalis*, indicating that MHC polymorphism has been maintained over evolutionary time, perhaps due to pathogen-mediated processes. We have yet to link selection with response to a particular pathogen; nevertheless, two recent studies have directly highlighted the utility of MHC molecular markers in assessing population responses to disease in European species. Teacher et al. [19] found an association between MHC class I alleles and ranavirus infection status in *R. temporaria*. In addition, May et al. [20] found that MHC class II genotype frequencies in wild toad populations may have experienced directional selection in response to *Bd* introduction. The present study provides resources that can be used to perform these types of studies on other ranid species to inform management and conservation practices.

## Notes

**Acknowledgements: **We thank Duma Dániel, Bartha Csaba and Bartha László for help with specimen collection and Jakab Endre and Chira Sergiu for assistance with molecular protocols.
